# Myeloid Sarcoma of Orbits: Effectiveness of a Low-Dose Radiation Regimen

**DOI:** 10.1155/2018/9071693

**Published:** 2018-09-09

**Authors:** Shyam Ravisankar, Yair Levy, Maya Shah

**Affiliations:** ^1^Division of Hematology and Oncology, Newark Beth Israel Medical Center, Newark, NJ, USA; ^2^Department of Radiology, Newark Beth Israel Medical Center, Newark, NJ, USA

## Abstract

Acute myeloid leukemia (AML) can present with extramedullary involvement known as myeloid sarcoma (MS). We present the case of a young woman who was diagnosed with AML and MS in bilateral orbits, brain, omentum, and retroperitoneum. She was treated with induction chemotherapy. Low-dose radiation was given to the orbits due to visual symptoms which resulted in complete response. The use of radiation therapy in orbital MS has not been studied extensively, and low dose may be adequate to achieve complete remission (CR) in selected patients.

## 1. Background

Acute myeloid leukemia (AML) can present with extramedullary (EM) involvement as granulocytic sarcoma also known as chloroma or myeloid sarcoma (MS). MS can present as an isolated extramedullary leukemic tumor, concurrently with or at relapse of acute myeloid leukemia. We present the case of a young female who had de novo AML with MS in bilateral orbits, brain, omentum, and retroperitoneum. The use of radiation therapy (RT) in orbital MS has not been studied extensively, and this presents a therapeutic dilemma.

## 2. Case Presentation

**Figure 1:**
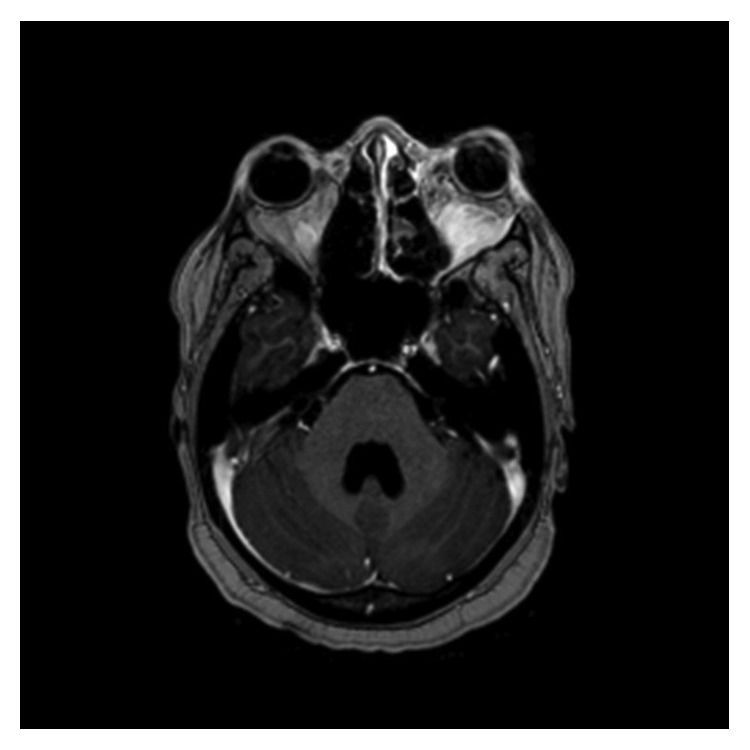


**Figure 2:**
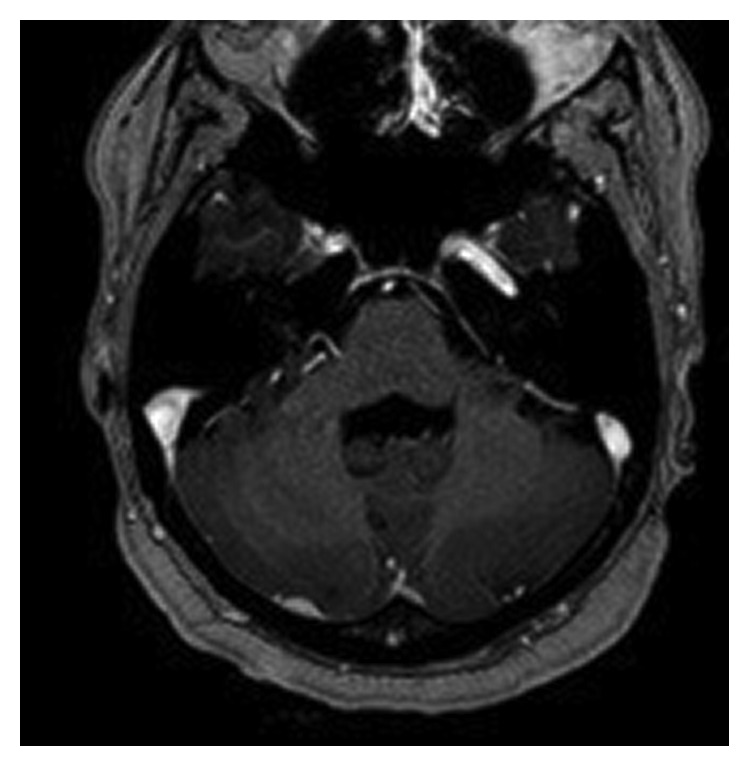


**Figure 3:**
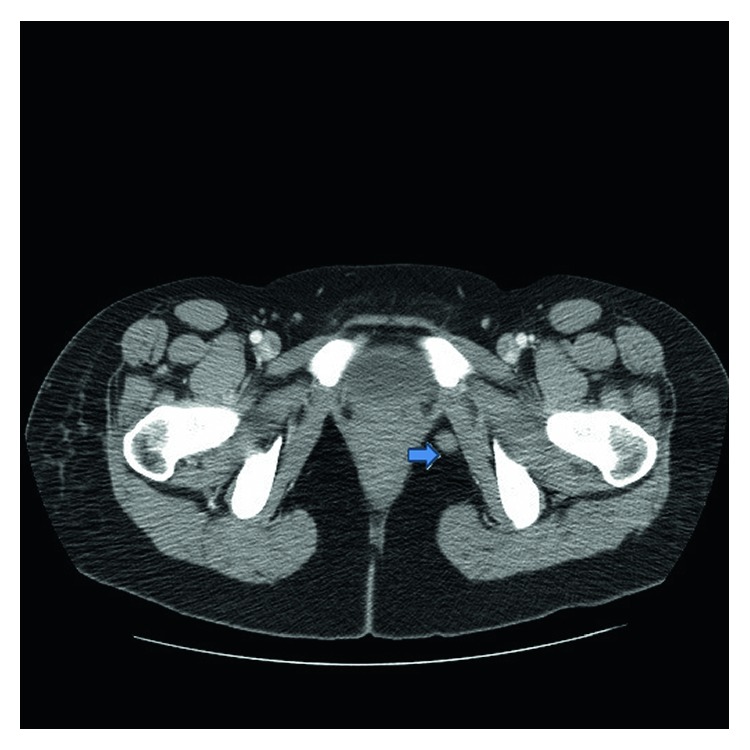


A 31-year-old African American female was found to have severe anemia on laboratory work performed by her gynecologist. The patient reported fatigue and dyspnea on exertion for about two months. She also had a two-week history of gum bleeding while brushing her teeth. She denied any other bleeding, weight loss, chills, or fevers. She had occasional blurry vision for about two weeks. Physical examination was unremarkable except for proptosis of the left eye. Visual fields and acuity were normal. A complete blood count showed a white blood cell count of 18 × 10^9^/L with 50% blasts, hemoglobin of 5.7 g/dl, and a platelet count of 18 × 10^9^/L. Bone marrow aspirate showed myeloblasts (64.2%) by flow cytometry. The myeloblasts were positive for CD13, CD33, CD34, MPO, and HLA-DR and negative for CD20 and CD19 on immunophenotyping. Fluorescent in situ hybridization (FISH) analysis revealed translocation t(8;21). Testing for FLT3 (Fms-like tyrosine kinase 3), CEBPA (CCAAT/enhancer-binding protein alpha) and NPM1 (nucleophosmin 1) was negative. Cerebrospinal fluid analysis was negative for involvement by leukemia. MRI of the brain demonstrated bilateral orbital masses measuring 2.6 cm on the right and 1.2 cm on the left (Figure 1). The left orbital mass spanned the intraconal and extraconal compartments and displaced the optic nerve superomedially. The right orbital mass was in the right orbital apex extending along the roof of the orbit. Both masses were separate from the optic nerve and the ocular globe. Both masses compressed the optic nerve at the apex. There was mild proptosis on the left and only minimal proptosis on the right. There was an enhancing dural-based lesion in the right posterior fossa measuring 11 × 3 mm (Figure 2). Nodules measuring around one centimeter were noted in the omentum, retroperitoneal space, and the left ischiorectal fossa on computed tomography (CT) scan of the abdomen (Figure 3).

**Figure 4:**
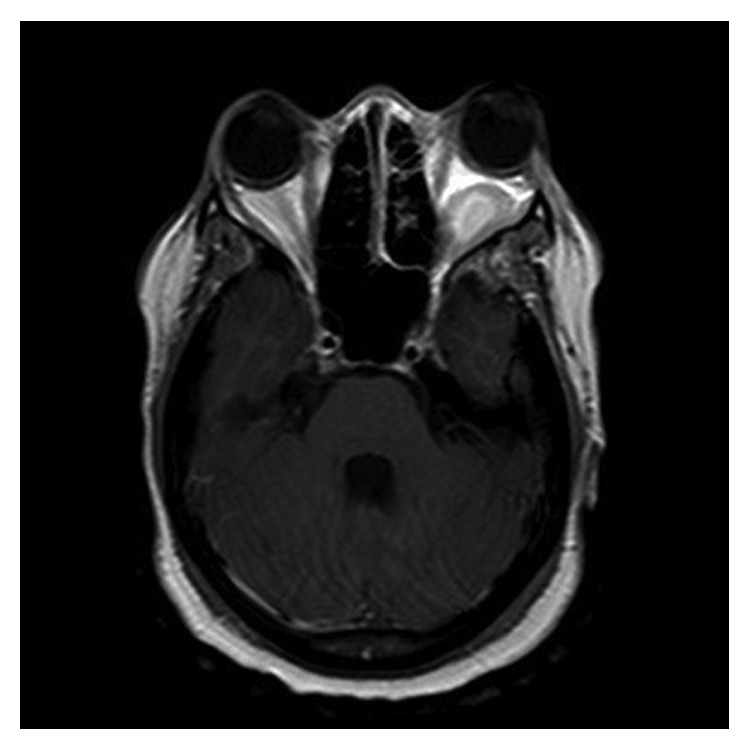


The patient was treated with cytarabine 200 mg/m^2^ and daunorubicin 90 mg/m^2^ (7 + 3 regimen) induction chemotherapy. Radiotherapy (RT) to the orbital MS was administered due to visual symptoms. The first treatment was on day 2 of chemotherapy. The treatment consisted of 50 cGy fractions using opposed lateral fields and half-beam blocks using 6 mV photons to both orbits. The planning was done in such a way that the lens was not in the radiation field, and the conus was included in the radiation field. Two days later, MRI of the brain showed interval resolution of the enhancing mass in the right orbital apex, decreased proptosis of the left eye secondary to decreased size, and enhancement of the orbital mass which had diminished in size to 1.5 × 1.0 cm compared to 2.6 × 1.9 cm on the prior scan (Figure 4). The dural-based mass in posterior fossa had nearly resolved. The patient had an improvement in blurry vision after two doses of RT. The patient received a total of 150 cGy to the right orbit and 200 cGy to the left orbit in four fractions over a week. The RT treatment details are shown in Table 1. There were no acute toxicities of RT. Complete hematologic and cytogenetic response was achieved on day 28 after the induction chemotherapy.

**Figure 5:**
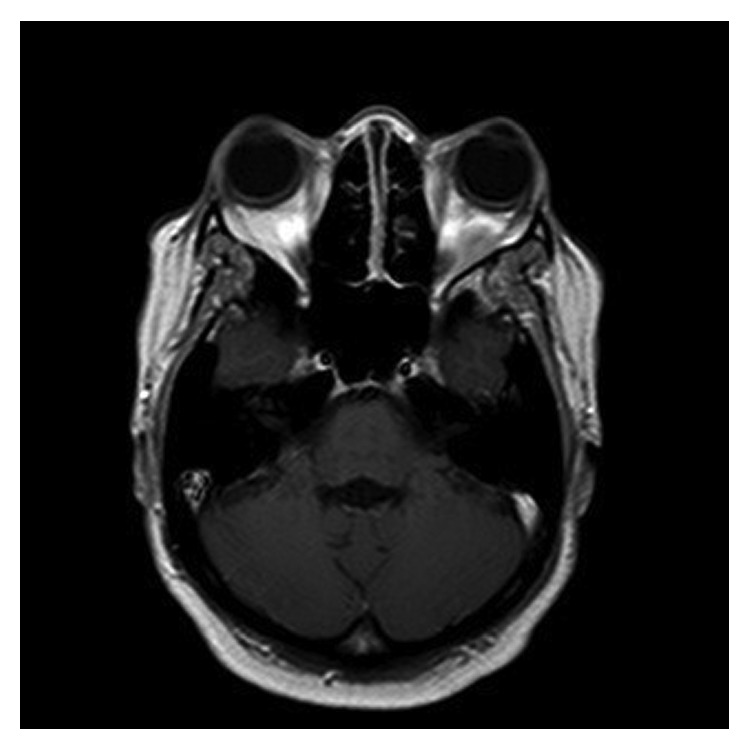


**Figure 6:**
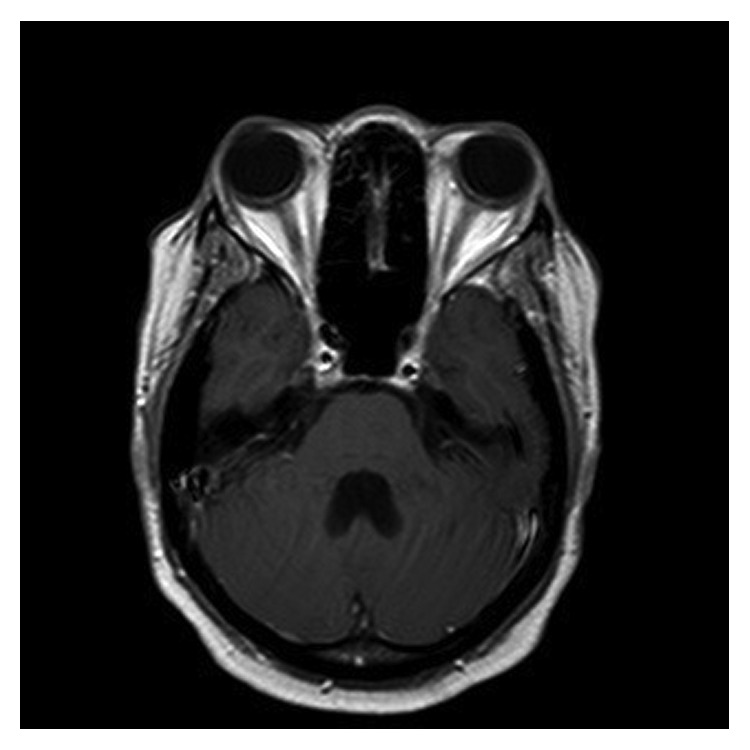


**Figure 7:**
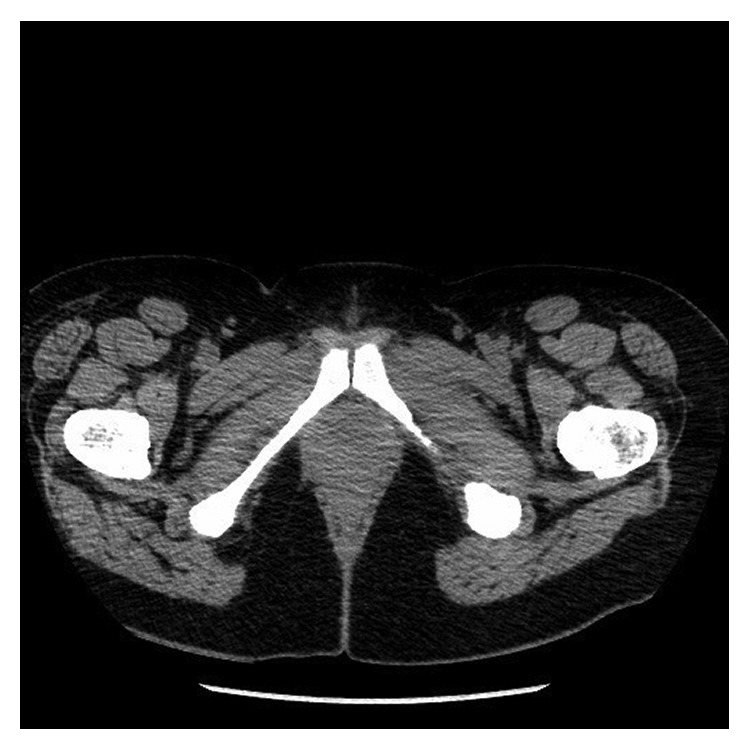


One month later, MRI of the brain showed a decrease in enhancement of the left orbital mass likely representing residual scar and complete resolution of MS in the right orbit and brain (Figure 5). Her visual symptoms and proptosis had resolved. There was also resolution of the dural-based posterior fossa lesion (Figure 6). CT scan of the abdomen and pelvis showed resolution of all the nodules in the abdomen and pelvis (Figure 7). She was evaluated for stem cell transplantation and was deemed to have low-risk disease. The patient proceeded to receive consolidation chemotherapy with high-dose cytarabine for four cycles. The patient remained in complete remission eighteen months since diagnosis. No chronic adverse effects of RT were reported.

## 3. Discussion

Myeloid sarcoma (MS) is a rare extramedullary (EM) tumor of immature myeloid cells, often referred to as chloroma because of its green color caused by the presence of myeloperoxidase (MPO) [1]. Primary MS may occur de novo in the absence of any past or current bone marrow involvement by acute myeloid leukemia (AML), myelodysplastic syndrome (MDS), or myeloproliferative disorder (MPD) [2]. The primary form of MS is relatively rare. On the other hand, secondary MS (associated with marrow involvement) occurs in approximately 1.4% to 9% of patients with AML [2, 3].

MS is most commonly reported in the skin, bone, and lymph nodes. It can however involve many other sites such as the central nervous system (CNS), oral and nasal mucosa, breast, genitourinary tract, chest wall, pleura, retroperitoneum, gastrointestinal tract, and testis [4, 5]. Orbital involvement is very rare in adults though commonly seen in children [6, 7]. The most common manifestation of MS is compressive symptoms involving the adjacent structures. It can also present with pain and bleeding. In the orbits, the most common symptom is proptosis. Other symptoms may include headache, photophobia, diplopia, and intermittent eye pain depending on the size of the lesions and the extent of involvement of other structures [8].

MS can be difficult to diagnose based on imaging alone especially in cases of primary MS. It can be mistaken for an abscess, hemorrhage, or even lymphoma. Imaging features, particularly of central nervous system MS, that help to distinguish these lesions from other common complications of leukemia include multiple enhancing solid masses occurring at different sites. One caveat is that a mass with an enhancing peripheral rim and a hypodense or hypointense center may be indistinguishable from an abscess. In such cases, aspiration biopsy is necessary for a definitive diagnosis. The imaging modalities commonly used are CT, magnetic resonance imaging (MRI), and FDG positron emission tomography (PET) scan. If there is bone marrow involvement, a biopsy may not be necessary for diagnosis of MS.

MS has been reported in association with a variety of chromosomal abnormalities. In particular, t(8;21)(q22;q22) has been identified in reported cases of MS occurring more commonly in childhood and/or at the orbital level [9, 10].

At initial presentation, patients with isolated MS without bone marrow involvement as well as those with AML should be treated with systemic chemotherapy. Radiation (RT) is used if there is residual disease after chemotherapy or as consolidation treatment. Surgical intervention can be considered if there is rapid enlargement of MS with significant impairment of function, especially in the CNS and orbits.

RT is typically used for CNS lesions. It can also be used for other sites if they are causing symptoms or do not resolve after systemic chemotherapy. RT was studied in a retrospective analysis of thirty eight patients who underwent treatment for MS at a single institution [11]. It should be noted that there were no patients with orbital MS in the study. It was found that RT resulted in excellent local disease control and palliation of symptoms without significant toxicity. The study authors recommended irradiating MS to at least 20 Gy and proposed 24 Gy in 12 fractions as an appropriate regimen.

In another single-institution study of twenty patients, clinicopathologic features and responses to RT of MS were studied which revealed that the CR rate was optimal using RT doses between 20 Gy and 30 Gy with conventional fractionation. 97% of the patients achieved good local control of MS. Younger age, BMT prior to RT, and AML had a higher rate of CR. It is important to note that CR rate was not statistically different between the patients who received RT dose less than 22 Gy versus greater than 22 Gy. There were no patients with orbital MS in this study as well. RT to the orbits can lead to both acute and chronic toxicities. Erythema, conjunctivitis, keratitis, corneal ulceration, iritis, and retinal edema are some of the acute adverse effects of RT. Tissue necrosis, decreased tear production, telangiectasia, scleral melting, neovascularization, retinopathy, and secondary cancers can occur as long-term sequelae of orbital RT.

In our patient with bilateral orbital MS, RT at a low dose of 200 cGy was adequate to achieve CR. MRI after the first dose of RT showed a good response with complete resolution of the right orbital lesion and a 50% reduction of the left orbital lesion. It should be noted that the patient was receiving systemic chemotherapy in addition to RT. Based on the rapid response to therapy, a short course of low-dose RT was planned to avoid potential adverse effects to the eyes. We propose that the RT dose in patients with orbital MS should be individualized based on the size and location of the lesions. It may be valuable to obtain MRI or CT scan depending on the location of MS after the first-dose RT to evaluate the response and tailor the RT treatment plan.

## 4. Conclusion

MS is a rare extramedullary manifestation of AML. Regardless of the location, it is considered a systemic disease and should be treated with systemic chemotherapy. Radiation therapy in addition to systemic chemotherapy has been used with success to control MS. Low dose and short course of RT may be sufficient to achieve CR in selected patients with symptomatic orbital involvement who received systemic chemotherapy.

## Figures and Tables

**Table 1:** 

RT	Right orbit (cGy)	Left orbit (cGy)
Dose 1	50	50
Dose 2	50	50
Dose 3	50	50
Dose 4	0	50
Total dose	150	200

## References

[1] Brunnung R. D., Matutes E., Flandrin G., Jaffe E. S., Harris N. L., Stein H., Vardiman J. W. Acute myeloid leukemias. *Pathology and Genetics of Tumors of Haematopoietic and Lymphoid Tissue*.

[2] Tsimberidou A. M., Kantarjian H. M., Estey E. (2003). Outcome in patients with nonleukemic granulocytic sarcoma treated with chemotherapy with or without radiotherapy. *Leukemia*.

[3] Neiman R. S., Barcos M., Berard C. (1981). Granulocytic sarcoma: a clinicopathologic study of 61 biopsied cases. *Cancer*.

[4] Pileri S. A., Ascani S., Cox M. (2007). Myeloid sarcoma: clinicopathologic, phenotypic and cytogenetic analysis of 92 adult patients. *Leukemia*.

[5] Al-Khateeb H., Badheeb A., Haddad H., Marei L., Abbasi S. (2011). Myeloid sarcoma: clinicopathologic, cytogenetic, and outcome analysis of 21 adult patients. *Leukemia Research and Treatment*.

[6] Dinand V., Yadav S. P., Grover A. K., Bhalla S., Sachdeva A. (2013). Orbital myeloid sarcoma presenting as massive proptosis. *Hematology/Oncology and Stem Cell Therapy*.

[7] Payne C., Olivero W. C., Wang B. (2014). Myeloid sarcoma: a rare case of an orbital mass mimicking orbital pseudotumor requiring neurosurgical intervention. *Case Reports in Neurological Medicine*.

[8] Schwyzer R., Sherman G. G., Cohn R. J., Poole J. E., Willem P. (1998). Granulocytic sarcoma in children with acute myeloblastic leukemia and t(8;21). *Medical and Pediatric Oncology*.

[9] Rubnitz J. E., Raimondi S. C., Halbert A. R. (2002). Characteristics and outcome of t(8;21)-positive childhood acute myeloid leukemia: a single institution’s experience. *Leukemia*.

[10] Bakst R., Wolden S., Yahalom J. (2012). Radiation therapy for chloroma (granulocytic sarcoma). *International Journal of Radiation Oncology, Biology, Physics*.

[11] Chen W. Y., Wang C. W., Chang C. H. (2013). Clinicopathologic features and responses to radiotherapy of myeloid sarcoma. *Radiation Oncology*.

